# HYPERGLYCEMIA AND INCIDENCE OF FRAILTY IN OLDER MEXICAN ADULTS LIVING IN RURAL AREAS

**DOI:** 10.1093/geroni/igz038.2511

**Published:** 2019-11-08

**Authors:** Betty Manrique Espinoza, Ana Rivera Almaraz, Aaron Salinas Rodriguez

**Affiliations:** National Institute of Public Health, Cuernavaca, Morelos, Mexico

## Abstract

Hyperglycemia is the main characteristic of diabetes and is the result of an absolute or partial deficit in the production or action of insulin. Recent evidence suggests that hyperglycemia increases the risk of frailty. This issue is of great importance for the Mexican population given the high prevalence of diabetes, particularly in older adults. Our objective was to analyze the association between hyperglycemia and the incidence of frailty in a cohort of rural older adults in Mexico. Prospective cohort study with 600 rural older adults, with measurements made in 2009, 2013 and 2018. Fragility was defined using the proposal of Fried and colleagues. The determination of glycosylated hemoglobin was performed through the A1CNow® device, with capillary blood; hyperglycemia was defined considering the recommendation of the American Diabetes Association; where values greater than 6.5% (140 mg / dL) of glycosylated hemoglobin were considered hyperglycemia. We used an ordinal logistic regression model to analyze the relationship between hyperglycemia and incidence of frailty. In the baseline measurement (2009), 8.6% of older adults presented frailty. The incidence of frailty was 6.9%. After adjusting for health and sociodemographic characteristics, hyperglycemia was significantly associated with the incidence of frailty (RR = 2.24 P = 0.018). These findings allow us to determine that hyperglycemia is a prognostic factor of the incidence of frailty. Because frailty is preventable, future interventions for the prevention of frailty should consider the presence of hyperglycemia.

